# Urinary SARS-CoV-2 RNA Is an Indicator for the Progression and Prognosis of COVID-19

**DOI:** 10.3390/diagnostics11112089

**Published:** 2021-11-12

**Authors:** Lu Zhang, Maoqing Tian, Yuan Song, Wei Liang, Xiaogang Li, Yongqing Tong, Huiming Wang

**Affiliations:** 1 Department of Nephrology, Renmin Hospital of Wuhan University, Wuhan 430060, China; zhanglu.1@foxmail.com (L.Z.); tianmq95@163.com (M.T.); bogehers@163.com (Y.S.); Dr.liangwei@whu.edu.cn (W.L.); 2 Division of Nephrology, Department of Medicine, Mayo Clinic, Rochester, MN 55905, USA; Li.Xiaogang@mayo.edu; 3 Department of Laboratory Science, Renmin Hospital of Wuhan University, Wuhan 430060, China

**Keywords:** COVID-19, SARS-CoV-2, endothelium, renal pathology, indicator

## Abstract

Background: We aimed to analyze clinical characteristics and find potential factors to predict poor prognosis in patients with coronavirus disease 2019 (COVID-19). Methods: We analyzed the clinical characteristics and laboratory tests of COVID-19 patients and detected SARS-CoV-2 RNA in urine sediments collected from 53 COVID-19 patients enrolled in Renmin Hospital of Wuhan University from 31 January 2020 to 18 February 2020 with qRT-PCR analysis. Then, we classified those patients based on clinical conditions (severe or non-severe syndrome) and urinary SARS-CoV-2 RNA (U_RNA_^−^ or U_RNA_^+^). Results: We found that COVID-19 patients with severe syndrome (severe patients) showed significantly higher positive rate (11 of 23, 47.8%) of urinary SARS-CoV-2 RNA than non-severe patients (4 of 30, 13.3%, *p* = 0.006). U_RNA_^+^ patients or severe U_RNA_^+^ subgroup exhibited higher prevalence of inflammation and immune discord, cardiovascular diseases, liver damage and renal dysfunction, and higher risk of death than U_RNA_^−^ patients. To understand the potential mechanisms underlying the viral urine shedding, we performed renal histopathological analysis on postmortems of patients with COVID-19 and found severe renal vascular endothelium lesion characterized by an increase of the expression of thrombomodulin and von Willebrand factor, markers to assess the endothelium dysfunction. We proposed a theoretical and mathematic model to depict the potential factors that determine the urine shedding of SARS-CoV-2. Conclusions: This study indicated that urinary SARS-CoV-2 RNA detected in urine specimens can be used to predict the progression and prognosis of COVID-19 severity.

## 1. Introduction

COVID-19 is a highly contagious disease caused by a newly emerging coronavirus called severe acute respiratory syndrome coronavirus 2 (SARS-CoV-2), which belongs to the β-coronavirus cluster that comprises 7 members, including SARS and Middle East respiratory syndrome (MERS) [1]. COVID-19 affects different people in different ways. SARS-CoV-2 infected patients have a wide range of symptoms reported—from mild symptoms to severe illness. In general, COVID-19 patients have usually demonstrated respiratory system symptoms, abnormal radiological images of chest computed tomography (CT) scan, and hematological changes [2]. Those patients under severe conditions might also suffer multiple organ damage, such as on kidney, heart, digestive tract, blood and nervous system [3]. The main methods used for screening and diagnosis of SARS-CoV-2 infection include the detection of SARS-CoV-2 nucleic acid, SARS-CoV-2-specific antibody and antigen [4]. Antigen-based diagnostic tests are less sensitive than reverse transcription-polymerase chain reaction (RT-PCR) -based tests, but they are a faster and more convenient alternative to PCR [5]. The test for SARS-CoV-2 nucleic acid in nasopharyngeal swab specimens with RT-PCR was almost the only pathogen detection method used at the early stage of the COVID-19 outbreak in Wuhan, China, and later this test was applied to other body fluid samples, such as anal swab, stool, blood and urine, to avoid false negative results. It has been demonstrated that the positive rates of SARS-CoV-2 nucleic acid in different body fluids are variable, indicating a distinct pattern of persistence and clearance of viral RNA in body fluids in COVID-19 patients [6,7,8]. The positive rates detected in extra-pulmonary specimens are usually lower than those detected in nasopharyngeal swabs, which may have special significance in the evaluation of disease condition and determining the virus shedding routes [6,7]. Similar to previous reports [6,7,8,9,10,11,12,13,14,15], we also found that urinary SARS-CoV-2 RNA could be detected in COVID-19 patients, indicating that under certain specific conditions SARS-CoV-2 might be infiltrated from blood stream to kidney parenchyma, and eventually resulted in renal injury and urinary shedding of viruses. Given the acute kidney injury (AKI) is a common complication among hospitalized patients with severe COVID-19 infection [16,17,18], coexisting with a low urinary virus RNA positive rate in COVID-19 patients. We hypothesize that the detection of urinary SARS-CoV-2 nucleic acid, which may result in renal and cardiovascular endothelial destruction to facilitate the virus access to the kidney parenchyma, with the improved method may be used as a specific biomarker to indicate the severity of COVID-19.

## 2. Materials and Methods

### 2.1. Study Design and Patients

From 31 January 2020 to 18 February 2020, a total of 53 patients who were diagnosed with COVID-19 at Renmin Hospital of Wuhan University were tested for SARS-CoV-2 nucleic acid in urine samples with quantitative reverse transcription-polymerase chain reaction (qRT-PCR) analysis. Patients with pre-existing kidney disease were excluded from this study. To reduce false negative results, we collected the urine sediment samples from those patients at the admission day case by case. Based on the results of urine SARS-CoV-2 nucleic acid testing, we divided those patients into two groups, including the urinary SARS-CoV-2 negative group (U_RNA_^−^, 38 cases) and positive group (U_RNA_^+^, 15 cases). We then conducted a retrospective study on those patients’ clinical characteristics, pre-existing diseases and laboratory tests (Figure S1). The diagnosis of COVID-19 pneumonia was conducted by following the New Coronavirus Pneumonia Prevention and Control Guidance (5th edition) published by the National Health Commission of China [19]. Our study was approved by the ethics committee of Renmin Hospital of Wuhan University (wdry2020-k064), the Ethics Commission of General Hospital of Central Theatre Command ([2020]017-1), and the Ethics Commission of Jinyintan Hospital (KY-2020-15.01). Written informed consent was waived by the Ethics Commission of the participated hospitals for emerging infectious diseases.

### 2.2. Data Collection

The data of epidemiological characteristics, clinical manifestation, radiology examination and laboratory examination were collected from the electronic medical records, and the laboratory examination included arterial blood gas test, myocardial enzyme, heart failure, whole blood cell count, liver and kidney function, electrolytes, blood lipid, coagulation test, immunoglobulin, complement and C-reactive protein. The illness conditions were assessed and defined as severe and non-severe type depending on the existence of respiratory dysfunction, in that the severe type was defined as the oxygen saturation being less than 93% under resting status, or the arterial oxygen pressure (PaO_2_)/fraction of inspired oxygen (FiO_2_) ratio is less than 300 mmHg. We identified 30 cases as non-severe patients and 23 as severe patients (Figure S1). All data were reviewed by a team of physicians.

### 2.3. Virological Analysis

The SARS-CoV-2 virus in urine from the 53 COVID-19 patients was detected with quantitative RT-PCR analysis as previously described [20]. In brief, the urine sediments from participants were collected for SARS-CoV-2 test with the detection kit (Bioperfectus, Taizhou, China). The ORF1ab gene (nCovORF1ab) and the N gene (nCoV-NP) were used for qRT-PCR analysis according to the manufacturer’s instructions. Reaction mixtures were prepared and qRT-PCR assay was then performed under the following conditions: incubation at 50 °C for 15 min and 95 °C for 5 min, 40 cycles of denaturation at 94 °C for 15 s, and extending and collecting fluorescence signal at 55 °C for 45 s.

### 2.4. Tissue Sampling and Processing

Kidney samples were obtained from autopsies of 4 severe COVID-19 patients with multi-organ failure, including acute kidney injury (AKI). The histopathological of AKI, including tubular luminal dilatation, simplification of the lining epithelium, loss of epithelial cell nuclei in some cells and loss of the brush border, and/or tubular epithelial cell necrosis. Renal histopathology was examined in a designated laboratory.

Immunohistochemical staining was performed on kidney specimens from autopsy for thrombomodulin (TM), and von Willebrand factor (vWF) as previously described [21]. Briefly, the sections were incubated with primary anti-TM (Cat: 14318-1-AP, 1:100, rabbit IgG; Proteintech Group, Rosemont, IL, USA), anti-vWF (Cat: 11778-1-AP, 1:100, rabbit IgG; Proteintech Group, Rosemont, IL, USA), or rabbit-isotype antibody (control) (1:100; Dako) at 4 °C overnight, followed by the incubation with the HRP-anti-Rabbit secondary antibodies for 1 h at room temperature. Peroxidase activity was visualized with the DAB Elite kit (K3465, DAKO). Positive staining as brown coloration was viewed by a light microscope.

### 2.5. Statistical Analysis

Continuous variables were expressed using the mean ± standard deviation (normal distribution) or medians and interquartile (IQR) values as appropriate (abnormal distribution). Categorical variables were shown as the percentages and counts. Two-independent group *t*-tests was used when the data were normally distributed, otherwise, Wilcoxon rank-sum test was used. Chi-square tests and Fisher’s exact tests were applied to categorical variables as appropriate. The cumulative rate of in-hospital survival was investigated using the Kaplan–Meier method. All statistical analyses were performed using SPSS 22.0 (Chicago, IL, USA). *p* < 0.05 was considered as statistically significant.

## 3. Results

### 3.1. Characterization of Patients with U_RNA_^+^ and U_RNA_^−^

**Figure 1:**
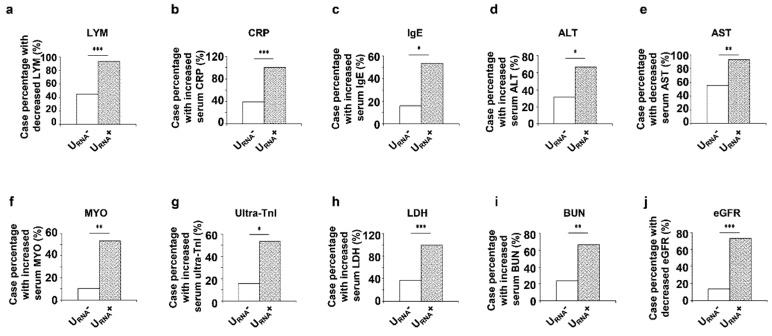
Categorical variable results of laboratory tests of COVID-19 patients on admission. (**a**–**j**) The case percentage of decreased serum LYM (**a**), and increased serum CRP (**b**), IgE (**c**), ALT (**d**), decreased serum AST (**e**), increased serum MYO (**f**), ultra-TnI (**g**), LDH (**h**), BUN (**i**), and decrease eGFR (**j**) in U_RNA_^−^ and U_RNA_^+^ patients. LYM: lymphocyte; MYO: myoglobin; ultra-TnI: cardiac troponin I; * *p* < 0.05, ** *p* < 0.01, *** *p* < 0.001.

A total of 53 hospitalized COVID-19 patients were enrolled in this study. The characteristics of those patients were detailed in Table 1. The median age of those patients was 52 years old (IQR, 42–66), and 58% of those patients were female. By testing SARS-CoV-2 nucleic acid in urine samples with qRT-PCR analysis, we found that 38 of those 53 patients were urinary SARS-CoV-2 negative (U_RNA_^−^). The urinary SARS-CoV-2 positive (U_RNA_^+^) patients were older and more likely to experience chest tightness and shortness of breath than U_RNA_^−^ patients, but showed no significant differences in male/female distribution, fever, cough, sputum production, fatigue, radiological appearance, hypertension, diabetes, cardiovascular diseases, chronic renal disease (Table 1). In addition, U_RNA_^+^ patients suffered more severe respiratory distress with manifestations of lower arterial oxygen pressure (PaO_2_) and oxygen saturation (SaO_2_) than U_RNA_^−^ patients as examined with arterial blood gas analysis (Table 2). The leukopenia and lymphocytopenia were detected more frequently in routine blood test of U_RNA_^+^ patients than those in blood test of U_RNA_^−^ patients, (*p* < 0.001, Figure 1a). Immune profile evaluation identified a more frequent increase of serum CRP (*p* < 0.05) and IgE (*p* < 0.001) in U_RNA_^+^ patients (Figure 1b,c). In addition, we found that U_RNA_^+^ patients had higher prevalence of increased serum levels of ALT (*p* < 0.05, Figure 1d), higher percentage of increased serum AST (*p* < 0.01, Figure 1e), higher case percentage of increased serum myoglobin, *p* < 0.01, ultra-TnI (*p* < 0.05, Figure 1f,g), LDH (*p* < 0.001, Figure 1h), BUN (*p* < 0.01, Figure 1i), and decreased eGFR (*p* < 0.001, Figure 1j) than U_RNA_^−^ patients. These data indicated that U_RNA_^+^ patients had more severe lesions on organs of liver, heart, and kidney. We further found that U_RNA_^+^ patients showed significantly lower levels of T cells and T helper (Th) cells (*p* = 0.005) in peripheral blood mononuclear cells, and higher levels of serum CRP (*p* = 0.001), ALT (*p* = 0.02), but lower serum AST (*p* = 0.008), and higher levels of DBIL (*p* = 0.008), LDH (*p* = 0.001), BUN (*p* = 0.003), and significantly lower eGFR (*p* = 0.001) than U_RNA_^−^ patients (Table 3).

### 3.2. Clinical Features and Prognosis of Severe Patients with U_RNA_^+^ and U_RNA_^−^

**Figure 2:**
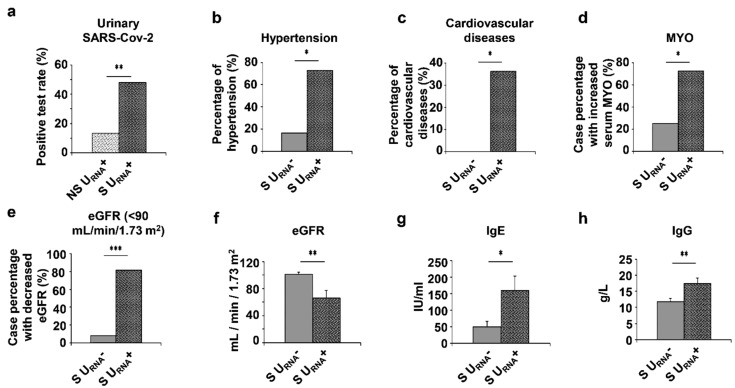
Categorical variable results of laboratory tests and comorbidities of COVID-19 patients on admission. (**a**) The positive test rate of urinary SARS-CoV-2 in non-severe (NS) U_RNA_+ and S U_RNA_+ patients. (**b**,**c**) The prevalence of hypertension and cardiovascular disease in Severe (S) U_RNA_^−^ and Severe U_RNA_+ patients. (**d**,**e**) The case percentage of increased serum MYO and decreased eGFR. in S U_RNA_^−^ and S U_RNA_^−^ patients. (**f**–**h**) The levels of eGFR, serum IgE and serum IgG in S U_RNA_^−^ and S U_RNA_^+^ patients. * *p* < 0.05, ** *p* < 0.01, *** *p* < 0.001.

**Figure 3:**
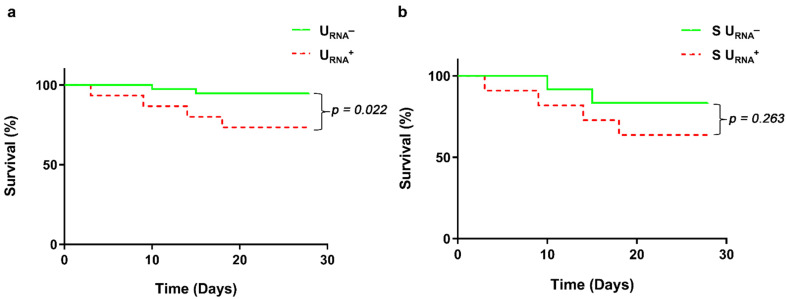
Survival curve for indicated groups of COVID-19 patients. (**a**) Survival curve for U_RNA_− and U_RNA_^+^ COVID-19 patients. Green solid line represents U_RNA_^−^ patients, and red dotted line represents U_RNA_+ patients. (**b**) Survival curve for server U_RNA_^−^ (S U_RNA_^−^) and S U_RNA_^+^ COVID-19 patients. Green solid line represents S U_RNA_− patients, and red dotted line represents S U_RNA_^+^ patients.

The above results implied a correlation of the urinary SARS-CoV-2 RNA with COVID-19 severity and the underlying conditions in COVID-19 patients. We hypothesized that urinary SARS-CoV-2 RNA may serve as a biomarker for predicting the clinical outcomes of severe COVID-19 patients. In the total of 53 patients, we identified 30 non-severe patients and 23 severe patients (Figure S1) based on the oxygen saturation (less than 93% under resting status) and the arterial oxygen pressure (PaO_2_)/fraction of inspired oxygen (FiO_2_) ratio (less than 300 mmHg). Within the 23 severe patients, we found that 12 of those 23 server patients were urinary SARS-CoV-2 negative (S U_RNA_^−^) and 11 of them were U_RNA_^+^ (S U_RNA_^+^). The positive rate of urine SARS-CoV-2 RNA were significantly higher in severe patients (11/23 severe patients = 47.8%) than that in non-severe patients (4/30 non-severe patients = 13.3%) (*p* < 0.01, Figure 2a). This result suggested that urine shedding SARS-CoV-2 correlated with the severity of the disease. To support this notion, we found that S U_RNA_^+^ patients experienced more comorbidities, including higher prevalence of hypertension (*p* < 0.05) and cardiovascular diseases (*p* < 0.05) (Figure 2b–d), and renal function impairment (*p* < 0.001) (Figure 2e). S U_RNA_^+^ patients also showed significantly lower levers of eGFR, *p* < 0.01 (Figure 2f) but higher levels of IgE (*p* < 0.05) and IgG (*p* < 0.01) (Figure 2g,h) than S U_RNA_^−^ patients. In addition, the U_RNA_^+^ patients among 53 patients had a significantly higher risk of death than the U_RNA_^−^ patients (*p* = 0.022, Figure 3a). Furthermore, the severe patients with U_RNA_+ also demonstrated a higher risk of death than the severe patients with U_RNA_^−^, although the difference did not reach statistical significance between U_RNA_^−^ and U_RNA_^+^ groups of severe patients (Figure 3b). 

### 3.3. The Expression of Thrombomodulin (TM) and von Willebrand Factor (vWF) Was Increased in Renal Tissues from Dead COVID-19 Patients

**Figure 4:**
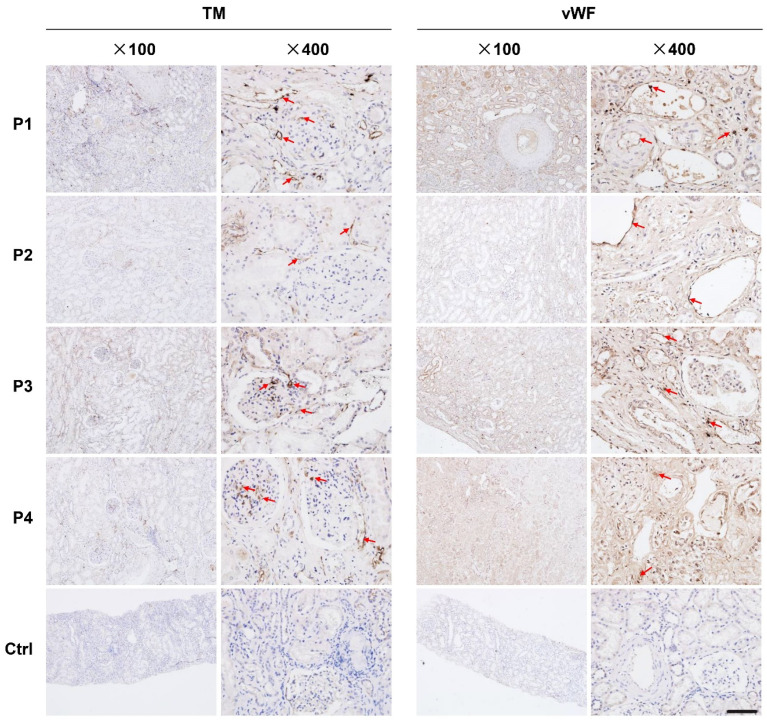
Histopathological examinations of endothelial lesion in kidney tissues. IHC staining of endothelial lesion markers of TM and vWF in kidney sections from dead COVID-19 patients after autopsy. TM and vWF expression in kidney tissues from four dead COVID-19 patients were observed under light microscope at 100- or 400-times magnification. Arrow indicated the positive staining cells. The adjacent renal tissue from the resected kidney from one patient with renal carcinoma was also stained as a control. TM: thrombomodulin; vWF: von Willebrand factor.

We found that the expression of TM and vWF in interstitial vessels, glomerular, and tubules were higher in kidneys from COVID-19 patients who had died compared to those in kidneys from renal carcinoma patients (Figure 4).

### 3.4. Theoretical and Mathematic Modeling of Urine Shedding of SARS-CoV-2

**Figure 5:**
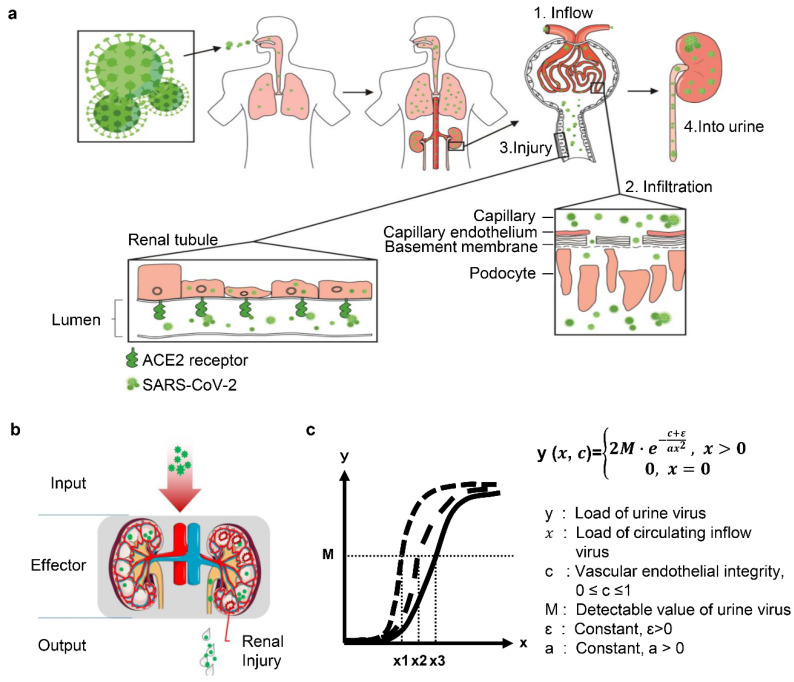
Theoretical and mathematical modeling of the event of urinary SARS-CoV-2 shedding. (**a**) Schematic diagram showed the renal inflow-infiltration-injury-into urine (“4I” model) to illustrate the course and outcome of SARS-CoV-2 infection in kidney. (**b**) Virus excretion can be simplified as a basic effector motif, consisting of input signal (inflow virus), effector (kidney) and output signal (urine shedding). (**c**) Function equation and curve of analytic mathematical model.

In this study, we adopt a model of renal inflow-infiltration-injury-into urine (“4I” model) to illustrate the course and outcome of SARS-CoV-2 infection in COVID-19 patient kidney (Figure 5a). In this model, the kidney is simplified as an effector to response to the virus invasion (input) with the effects of renal damages and virus shedding (output). Output effects are determined by the input strength (amount of loading virus) and the intrinsic nature of the kidney, such as the integrity of vascular endothelium and local immunity (Figure 5b). Although the compromised integrity of glomeruli and interstitial vascular vessels both allow the virus infiltration into kidney parenchyma, the infiltrating route through interstitial vessels is ignored, since the exuded virus is unlikely to infect tubular epithelial cells in which the ACE2 shed in the brush border [22]. In that context, we propose a function equation and curve to solve the urinary excretion of viral nucleic acid, condition of vascular endothelial integrity, and the circulating viral load in COVID-19 patients (Figure 5c).

## 4. Discussion

We conducted a comprehensive study on the clinical characteristics, pre-existing diseases and laboratory tests in a cohort of 53 COVID-19 patients, where 30 patients had non-severe symptoms, and 23 patients suffered from severe symptoms. Our results suggested an intriguing association of the urinary SARS-CoV-2 RNA with the clinical manifestation of COVID-19, pointing to a high viral load, new or pre-existing vascular endothelial damage in severe COVID-19 patients. This retrospective study indicated for the first time that the positive of SARS-CoV-2 RNA in urine specimens may be used to predict the progression and prognosis of COVID-19.

The SARS-CoV-2 presence in different body fluids, secretions, and excreta defines the infectious state of the patient. In addition to naso-/oro-pharyngeal swabs, the presence of SARS-CoV-2 RNA has also been reported in different biological samples such as feces, urine and blood. It is reported that feces contain viral RNA in a high percentage of cases and at a longer period of viral clearance [23]. Thus, it is important to improve our understanding of viral transmission through both respiratory and extra-respiratory routes in the management of these patients. It was reported that the positive rates of urine SARS-CoV-2 RNA vary from 0–7.5% (Table 4). This finding allows us to formulate a hypothesis: Viral infection and replication in kidney tissues indicate that the virus might be shed through urine. In the ferret model of SARS-CoV-2 infection, urinary SARS-CoV-2 RNA could be detected up to 8 days post-infection in the virus inoculated via the intranasal route group in contrast to only 4 days in direct contact ferrets [24], suggesting that the number of viral loads and the stages of illness may have an impact on the positive test rates. In addition, appropriate sampling seems to be essential for avoiding false negative results in COVID-19 patient samples. In our study, we optimized and analyzed the specimens of urine sediments, and found that 15 of those 53 patients were urinary SARS-CoV-2 positive (U_RNA_^+^) (Table 1). Our analysis showed a positive rate of 28.3% from 53 COVID-19 patients, which are higher than test results on routine urine sample testing (Table 4) [5,7,8,10,12,13]. The disparity in detection of the urinary SARS-CoV-2 RNA might have resulted in the false negative test of nucleic acid by qRT-PCR, which may be caused by inadequate sampling, low viral load, or other unknown factors [6,7]. This result suggested that our optimized urine SARS-CoV-2 RNA test method could improve the positive rate, which may assist in predicting the disease outcome, especially in severe patients. On the other hand, this information also indicated that recovering patients have a limited chance to spread the virus through urine.

We also observed that U_RNA_^+^ patients or severe U_RNA_^+^ subgroup showed higher prevalence of inflammation and immune dysfunction, cardiovascular diseases, liver damage and renal dysfunction, and higher risk of death than U_RNA_^−^ patients. The reason for the observed higher prevalence of developing severe clinical manifestations and higher risk of death in U_RNA_^+^ patients might be caused by a high SARS-CoV-2 viral load, which was shown to be strongly associated with in-hospital mortality in COVID-19 patients [25]. Endothelial dysfunction is prevalent in chronical cardiovascular disease and infectious or inflammatory diseases (such as that in virus-infected patients) [26,27,28,29,30,31,32]. TM and vWF have been recognized as biomarkers to assess the endothelium dysfunction [26,27,28,29,30]. Our data suggest that the new or preexisting vascular endothelial damage in COVID-19 patients may also lead to the increase of inflammation and damage within infected tissue and the excretion of SARS-CoV-2 into urine. Therefore, the evaluation of vascular endothelial damage may be an important prognostic tool to understand the outcomes of COVID-19 patients.

Studies on cellular mechanism have confirmed that SARS-CoV-2 shares the same membrane-bound angiotensin-converting enzyme 2 (ACE2) as SARS-CoV to gain access to its target cells [33,34,35]. In particular, kidneys show much more robust expression of ACE2 than respiratory organs, suggesting that kidney is a possible infecting target of SARS-CoV-2 [36]. The involvement of kidneys is usually evaluated in two aspects of renal functional impairment and renal insult. AKI has been reported as a severe complication of COVID-19 and it is associated with a heightened risk of mortality [37,38]. The reported incidence of AKI among hospitalized patients with COVID-19 varies widely, with ranges from 19–62.3% [18,38,39]. Chan et al. found that incidence for the patients admitted to ICU with AKI was up to 76% [40]. Kidney insults manifestations of proteinuria (44–84%) and hematuria (26.7–81%), were commonly seen in COVID-19 infection [3,17,40,41]. Until recently, the renal involvement in COVID-19 patients remains a matter of wide concern and debate as one study suggested that the renal insults were uncommon in COVID-19 patients [9]. To resolve the difference and controversy, two major issues need to be addressed. The first one is whether SARS-CoV-2 is able to infect the target cells through blood flow to kidney mesenchyme, and the second one is whether the infection can cause detrimental effects to the kidney tissue. Previous study has shown that spherical virus-like particles characteristic of coronavirus was found in the renal tubular epithelium and podocytes [42]. Considering that the infection of SARS-CoV-2 will trigger severe inflammation, we further explored whether SARS-CoV-2 infection induced renal endothelium dysfunction. We found that the expression of TM and vWF, biomarkers of endothelium dysfunction, were increased in renal tissues from dead COVID-19 patients (Figure 4). This result supported that SARS-CoV-2 can cause detrimental effects on kidney tissues. The factors related to the severity of renal dysfunction in COVID-19 patients are not yet fully understood. It could potentially be caused by the amount of infiltrated virus in kidneys, which may be largely dependent on the inflow virus and the nature of the endothelium.

SARS-CoV-2 mainly infects the host through the respiratory tract and spread to other organs or tissues through blood circulation. Damaged vascular endothelial cells, which either preexisted or was incurred by virus infection, allows virus infiltration between blood stream and tissue mesenchyme by passing through the endothelium. Our findings support a significant role of vascular endothelial lesion in COVID-19 patients in the development of renal damage and urine shedding. To further illustrate the course and outcome of SARS-CoV-2 infection in COVID-19 patient kidneys, we raised a model of renal inflow-infiltration-injury-into urine (“4I” model) (Figure 5a). This model may help us to understand the role and function of kidneys in SARS-CoV-2 infection. Kidneys are simplified as an effector to response to the virus invasion (input) with the effects of renal damage and virus shedding (output). SARS-CoV-2-related renal insult and urine shedding may be determined by several factors, including the amount of virus (viral load), the vascular endothelial integrity, as well as the intensity of anti-virus response. Our analysis on the clinical features of the patients showed that the positive test rate of urine SARS-CoV-2 RNA is remarkably higher in the severe patients than in non-severe patients, and renal vascular endothelial lesion is associated with severity of COVID-19 patients. Based on these finding, we propose a function equation and curve to solve the urinary excretion of viral nucleic acid, the condition of vascular endothelial integrity and the circulating viral load (Figure 5c). The function equation and curve may provide another strategy to calculate and predict the progression and prognosis of the disease.

However, due to the limited sample size, our model might not be powered sufficiently to reflect the overall complexity of the general population. Therefore, large-scale prospective cohort studies are required in ethnically and geographically diverse cohorts to better understand the association and importance of U_RNA_^+^ in the disease progression of COVID-19. In addition, due to the lack of clinical data of patients after discharge, we could not assess the association of urinary SARS-CoV-2 RNA with disease recovery. The precise relationship between urinary SARS-CoV-2 RNA and endothelial dysfunction and multiple organ dysfunction in these patients requires further investigation. Furthermore, our hypothesis about the function equation and curve to solve the urinary excretion of viral nucleic acid, the condition of vascular endothelial integrity and the circulating viral load, should be tested in future studies.

## 5. Conclusions

We optimized the method of urine SARS-CoV-2 RNA detection and significantly increased the positive detection rates. We analyzed the clinical characteristics of patients with urinary nucleic acid positive SARS-CoV-2 RNA, and revealed a potential association of vascular endothelial damage with virus urine shedding. Furthermore, we established a model to analyze the relationship between virus urine excretion and the underlying disease condition. In conclusion, this study suggests that the detection of SARS-CoV-2 RNA in urine sediments can provide a robust biomarker for evaluation and prognosis for patients with COVID-19.

## Figures and Tables

**Table 1:** Demographic and Clinical characteristics of 53 enrolled patients.

	All Patients (*n* = 53)	Urinary SARS-CoV-2		*p* Value	Illness Severity				*p* Value
		U_RNA_^−^ (*n* = 38, 71.7%)	U_RNA_^+^ (*n* = 15, 28.3%)	U_RNA_^−^ vs. U_RNA_^+^	Non-Severe		Severe		S U_RNA_^+^ vs. S U_RNA_^−^
					U_RNA_^−^ (*n* = 26, 86.7%)	U_RNA_^+^ (*n* = 4, 13.3%)	U_RNA_^−^ (*n* = 12, 52.2%)	U_RNA_^+^ (*n* = 11, 47.8%)	
Demographic characteristic (No., %)									
Age, years	52 (42.0–66.0)	51.0 (38.3–59.8)	61.0 (48.5–72.0)	0.016	50.5 (37.3–56.8)	50.0 (48.5–60.8)	55.0 (51.0–63.5)	66.0 (50.0–72.0)	0.126
Female	31/53 (58%)	22/38 (58%)	9/15 (60%)	0.889	17/26 (65%)	3/4 (75%)	5/12 (42%)	6/11 (55%)	0.684
Respiratory symptoms (No., %)									
Fever	41/53 (77.4%)	31/38 (73.7%)	13/15 (86.7%)	0.969	16/26 (61.5%)	4/4 (100.0%)	12/12 (100.0%)	9/11 (81.8%)	0.217
Cough	38/53 (71.7%)	31/38 (73.7%)	10/15 (66.7%)	0.421	19/26 (73.1%)	3/4 (75.0%)	9/12 (75.0%)	7/11 (63.6%)	0.667
Sputum production	13/53 (24.5%)	9/38 (23.7%)	6/15 (40.0%)	0.396	5/26 (19.2%)	3/4 (75.0%)	2/12 (16.7%)	3/11 (27.3%)	0.640
Fatigue	18/53 (34.0%)	11/38 (29.0%)	7/15 (46.7%)	0.220	9/26 (34.6%)	3/4 (75.0%)	2/12 (16.7%)	4/11 (36.4%)	0.371
Chest tightness	14/53 (26.4%)	6/38 (15.8%)	9/15 (60.0%)	0.001	3/26 (11.5%)	3/4 (75.0%)	2/12 (16.7%)	6/11 (54.6%)	0.089
Shortness of breath	19/53 (35.9%)	12/38 (31.6%)	10/15 (66.7%)	0.02	0/26 (0.0%)	0/4 (0.0%)	9/12 (75.0%)	10/11 (90.9%)	0.590
Radiological appearance (No., %)									
Unilateral pneumonia	2/53 (3.77%)	2/38 (5.3%)	0/15 (0.0%)	0.365	2/26 (7.7%)	0/4 (0.0%)	0/12 (0.0%)	0/11 (0.0%)	UTC
Bilateral pneumonia	35/53 (66.0%)	26/38 (68.4%)	9/15 (60.0%)	0.560	17/26 (62.4%)	1/4 (25.0%)	9/12 (75.0%)	8/11 (72.7%)	1.000
Multiple “ground-glass opacity” lesions	20/35 (37.7%)	17/38 (44.7%)	3/15 (20.0%)	0.094	14/26 (53.9%)	0/4 (0.0%)	3/12 (25.0%)	3/11 (27.3%)	1
Comorbidities (No., %)									
Hypertension	19/53 (35.9%)	11/38 (29.0%)	8/15 (53.3%)	0.095	9/26 (34.6%)	0/4 (0.0%)	2/12 (16.7%)	8/11 (72.7%)	0.012
Diabetes	7/53 (13.2%)	5/38 (13.2%)	2/15 (13.3%)	0.986	3/26 (11.5%)	0/4 (0.0%)	2/12 (16.7%)	2/11 (18.2%)	1.000
Cardiovascular diseases	6/53 (11.3%)	2/38 (5.3%)	4/15 (26.7%)	0.083	2/26 (7.7%)	0/4 (0.0%)	0/12 (0.0%)	4/11 (36.4%)	0.037
Chronic renal disease	2/53 (3.8%)	2/38 (5.3%)	0/15 (0.0%)	0.365	2/26 (7.7%)	0/4 (0.0%)	0/12 (0.0%)	0/11 (0.0%)	UTC
In-hospital death (No., %)	6/53 (11.3%)	2/38 (5.3%)	4/15 (26.7%)	0.083	0/26 (0.0%)	0/4 (0.0%)	2/12 (16.7%)	4/11 (36.4%)	0.371

Data shown as medians (interquartile ranges, IQR) and numbers/total (%); UTC, unable to calculate; U_RNA_^−^, negative urinary SARS-CoV-2; U_RNA_^+^, positive urinary SARS-CoV-2; S, Severe. *p* values presented the comparison between Negative cases and positive cases. *p* < 0.05 was considered statistically significant.

**Table 2:** Arterial blood gas analysis of 53 enrolled patients.

	All Patients (*n* = 53)	Urinary SARS-CoV-2		*p* Value	Illness Severity				*p* Value
		U_RNA_^−^ (*n* = 38)	U_RNA_^+^ (*n* = 15)	U_RNA_^−^ vs. U_RNA_^+^	Non-Severe		Severe		S U_RNA_^+^ vs. S U_RNA_^−^
					U_RNA_^−^ (*n* = 26)	U_RNA_^+^ (*n* = 4)	U_RNA_^−^ (*n* = 12)	U_RNA_^+^ (*n* = 11)	
Arterial blood gas (No., %)									
PO_2_ (<100)	18/53 (34.0%)	8/38 (21.1%)	10/15 (66.7%)	0.002	0/26 (0.0%)	0/4 (0.0%)	8/12 (66.7%)	10/11 (90.9%)	0.317
PO_2_ (<80)	13/53 (24.5%)	6/38 (15.8%)	7/15 (46.7%)	0.046	0/26 (0.0%)	0/4 (0.0%)	6/12 (50.0%)	7/11 (63.6)	0.680
PCO_2_ (>46)	9/53 (17.0%)	5/38 (13.2%)	4/15 (26.7%)	0.439	0/26 (0.0%)	0/4 (0.0%)	5/12 (41.7%)	4/11 (36.4%)	1.000
SaO_2_ (≤93)	23/53 (43.4%)	12/38 (31.6%)	11/15 (73.3%)	0.006	0/26 (0.0%)	0/4 (0.0%)	12/12 (100.0%)	11/11 (100.0%)	UTC

Data shown as numbers/total (%), *p* values present the comparison between Negative cases and positive cases. The normal ranges of PO_2_, PCO_2_ and SaO_2_ are 80–100 mmHg, 35–45 mmHg and >95%, respectively. UTC, unable to calculate; U_RNA_^−^, negative urinary SARS-CoV-2; U_RNA_^+^, positive urinary SARS-CoV-2; S, Severe.

**Table 3:** Continuous variable results of laboratory tests of COVID-19 patients on admission.

	All Patients (*n* = 53)	Urinary SARS-CoV-2		*p* Value
		U_RNA_^−^ (*n* = 38)	U_RNA_^+^ (*n* = 15)	U_RNA_^−^ vs. U_RNA_^+^
T cell	724.0 (353.0–1035.0)	809.9 (495.8–1123.5)	412.0 (213.5–800.5)	0.019
<723/mL	25/53 (47.2%)	16/38 (42.1%)	9/15 (60.0%)	0.240
Th cell	440.0 (189.0–709.0)	548.0 (219.0–747.8)	247.0 (128.5–349.0)	0.011
<404/mL	26/53 (49.1%)	14/38 (36.8%)	12/15 (80.0%)	0.005
CRP	16.6 (5.0–77.3)	5.0 (5.0–38.7)	77.3 (23.6–95.9)	0.022
>10 mg/L (No., %)	30/53 (56.6%)	15/38 (39.5%)	15/15 (100.0%)	0.001
ALT	31.0 (18.0–58.0)	27.0 (16.0–57.5)	52.0 (29.5–81.5)	0.029
>50 U/L (No., %)	22/53 (41.5%)	12/38 (31.6%)	10/15 (66.7%)	0.020
AST	1.19 (0.88–21.00))	1.50 (1.00–24.8)	0.95 (0.74–1.18)	0.001
<15 U/L (No., %)	35/53 (66.0%)	21/38 (55.3%)	14/15 (93.3%)	0.008
>40 U/L (No., %)	3/53 (5.7%)	2/38 (5.3%)	1/15 (6.7%)	1.000
DBIL	4.9 (2.7–7.4)	3.8 (2.2–5.5)	7.2 (5.0–8.7)	0.001
>8 mmol/L (No., %)	16/53 (30.2%)	7/38 (18.4%)	9/15 (60.0%)	0.008
LDH	276.0 (193.0–458.0)	206.5 (166.3–314.5)	443.0 (329.5–587.0)	0.001
>250 U/L (No., %)	29/53 (54.7%)	14/38 (36.8%)	15/15 (100.0%)	0.001
BUN	5.2 (4.0–8.8)	5.0 (3.7–6.4)	8.8 (4.1–11.7)	0.032
>8 mmol/L (No., %)	19/53 (35.8%)	9/38 (23.7%)	10/15 (66.7%)	0.003
eGFR	102.1 (86.3–115.6)	103.7 (93.6–119.5)	82.1 (63.7–99.2)	0.002
<90 mL/min/1.73 m^2^ (No., %)	16/53 (30.2%)	5/38 (13.2%)	11/15 (73.3%)	0.001

Data shown as medians (interquartile ranges, IQR) and numbers/total (%), *p* values presented the comparison between Negative cases and positive cases. UTC, unable to calculate; U_RNA_^−^, negative urinary SARS-CoV-2; U_RNA_^+^, positive urinary SARS-CoV-2; S, Severe. The normal ranges of T cell, Th cell, CRP, ALT, AST, DBIL, LDH, BUN and eGFR are 723–2737/μL, 404–1612/μL, <10 mg/L, 7–40 U/L, 13–35 U/L, 0–7 mmol/L, 120–250 U/L, 3.1–8 mmol/L and >90 mL/min/1.73 m^2^, respectively.

**Table 4:** Comparison of urinary SARS-CoV-2 nucleic acid detection in literature reports.

Authors	Sampling Method	Detecting Method	Positive Rate	Target Gene	Detection Kit	Participants Condition	Refs.
Huiming Wang, et al.	Urine sediments	RT-PCR	28.3%	NP and ORF1ab	Zhongzhi, Wuhan	30 non-severe 23 severe	This article
Luwen Wang, et al.	Urine sediments	RT-PCR	7.5%	NP and ORF1ab	Zhongzhi, Wuhan	48 non-CKD, 5 CKD	[11]
Chaolin Huang, et al.	Urine	RT-PCR	11%	NP and ORF1ab	ND	9 moderates	[2]
Hongzhou Lu, et al.	Urine	RT-PCR	6.9%	NP and ORF1ab	Master Biotechnology, China	Recovered	[7]
Zhenglin Yang, et al.	Urine	RT-PCR	0%	NP and ORF1ab	GeneoDx (GZ-TRM2, China), Maccura (Sichuan, China) and Liferiver (W-RR-0479-02, China)	5 Uncomplicated, 14 complicated	[9]
Barnaby Edward Young, et al	Urine	RT-PCR	0%	N, S, and ORF1ab	EZ1 virus mini kit v2.0 (Qiagen)	6 mild, 4 severe	[10]
Roman Wölfel, et al.	Urine	RT-PCR	0%	E- and RdRp	Tib-Molbiol, Berlin, Germany	mild	[12]
Chin Ion Lei, et al.	Urine	qRT-PCR	0%	NP and ORF1ab	BioGerm, China	2 mild, 4 moderates	[12]
Fujie Zhang, et al.	Urine	RT-PCR and ddPCR	0%	NP and ORF1ab	Shanghai BioGerm Medical Technology Co. LTD, China (RT-PCR) TargetingOne, Beijing, China (ddPCR)	ND	[13]

ND: not determined. Detecting Method: reverse transcription-polymerase chain reaction (RT-PCR); Positive Rate: positive rate of urinary SARS-CoV-2 RNA; Target Gene: Targeting SARS-CoV-2 genes.

## Data Availability

The data presented in this study are available on request from the corresponding author. The data are not publicly available due to privacy.

## Supplementary Materials

The following are available online at https://www.mdpi.com/article/10.3390/diagnostics11112089/s1, Figure S1: Study flowchart of patient’s enrolling and data analysis. In this study 53 hospital COVID-19 patients who underwent a urine sediment SARS-CoV-2 RNA test at the early stage of admission were enrolled. The patients were stratified according to illness severity and urinary qRT-PCR results. Demographic and clinical features were collected and analyzed.

## References

[1] Coronaviridae Study Group of the International Committee on Taxonomy of Viruses (2020). The species Severe acute respiratory syndrome-related coronavirus: Classifying 2019-nCoV and naming it SARS-CoV-19. Nat. Microbiol..

[2] Huang C., Wang Y., Li X., Ren L., Zhao J., Hu Y., Zhang L., Fan G., Xu J., Gu X. (2020). Clinical features of patients infected with 2019 novel coronavirus in Wuhan, China. Lancet.

[3] Wang D., Hu B., Hu C., Zhu F., Liu X., Zhang J., Wang B., Xiang H., Cheng Z., Xiong Y. (2020). Clinical characteristics of 138 hospitalized patients with 2019 Novel Coronavirus-infected pneumonia in Wuhan, China. JAMA.

[4] Peng Y.C., Cheng C.H., Yatsuda H., Liu S.H., Liu S.J., Kogai T., Kuo C.Y., Wang R.Y. (2021). A novel rapid test to detect Anti-SARS-CoV-2 N protein IgG based on shear horizontal surface acoustic wave (SH-SAW). Diagnostics.

[5] Spearman P. (2021). Diagnostic testing for SARS-CoV-2/COVID-19. Curr. Opin. Pediatr..

[6] Zhang W., Du R.-H., Li B., Zheng X.-S., Yang X.-L., Hu B., Wang Y.-Y., Xiao G.-F., Yan B., Shi Z.-L. (2020). Molecular and serological investigation of 2019-nCoV infected patients: Implication of multiple shedding routes. Emerg. Microbes Infect..

[7] Ling Y., Xu S.-B., Lin Y.-X., Tian D., Zhu Z.-Q., Dai F.-H., Wu F., Song Z.-G., Huang W., Chen J. (2020). Persistence and clearance of viral RNA in 2019 novel coronavirus disease rehabilitation patients. Chin. Med. J..

[8] Guan W.-J., Zhong N.-S. (2020). Clinical characteristics of Covid-19 in China. N. Engl. J. Med..

[9] Xie C., Jiang L., Huang G., Pu H., Gong B., Lin H., Ma S., Chen X., Long B., Si G. (2020). Comparison of different samples for 2019 novel coronavirus detection by nucleic acid amplification tests. Int. J. Infect. Dis..

[10] Young B.E., Ong S.W.X., Kalimuddin S., Low J.G., Tan S.Y., Loh J., Ng O.T., Marimuthu K., Ang L.W., Mak T.M. (2020). Epidemiologic features and clinical course of patients infected with SARS-CoV-2 in Singapore. JAMA.

[11] Wang L., Li X., Chen H., Yan S., Li D., Li Y., Gong Z. (2020). Coronavirus Disease 19 Infection does not result in acute kidney injury: An analysis of 116 hospitalized patients from Wuhan, China. Am. J. Nephrol..

[12] Wölfel R., Corman V.M., Guggemos W., Seilmaier M., Zange S., Müller M.A., Niemeyer D., Jones T.C., Vollmar P., Rothe C. (2020). Virological assessment of hospitalized patients with COVID-19. Nature.

[13] Newsome R.C., Gauthier J., Hernandez M.C., Abraham G.E., Robinson T.O., Williams H.B., Sloan M., Owings A., Laird H., Christian T. (2021). The gut microbiome of COVID-19 recovered patients returns to uninfected status in a minority-dominated United States cohort. Gut Microbes.

[14] Lo I.L., Lio C.F., Cheong H.H., Lei C.I., Cheong T.H., Zhong X., Tian Y., Sin N.N. (2020). Evaluation of SARS-CoV-2 RNA shedding in clinical specimens and clinical characteristics of 10 patients with COVID-19 in Macau. Int. J. Biol. Sci..

[15] Yu F., Yan L., Wang N., Yang S., Wang L., Tang Y., Gao G., Wang S., Ma C., Xie R. (2020). Quantitative detection and viral load analysis of SARS-CoV-2 in infected patients. Clin. Infect. Dis..

[16] Ronco C., Reis T. (2020). Kidney involvement in COVID-19 and rationale for extracorporeal therapies. Nat. Rev. Nephrol..

[17] Cheng Y., Luo R., Wang K., Zhang M., Wang Z., Dong L., Li J., Yao Y., Ge S., Xu G. (2020). Kidney disease is associated with in-hospital death of patients with COVID-19. Kidney Int..

[18] Ng J.H., Hirsch J.S., Hazzan A., Wanchoo R., Shah H.H., Malieckal D.A., Ross D.W., Sharma P., Sakhiya V., Fishbane S. (2021). Outcomes among patients hospitalized with COVID-19 and acute kidney injury. Am. J. Kidney Dis..

[19] (2020). New Coronavirus Pneumonia Prevention and Control Program.

[20] Wang M., Wu Q., Xu W., Qiao B., Wang J., Zheng H., Jiang S., Mei J., Wu Z., Deng Y. (2020). Clinical diagnosis of 8274 samples with 2019-novel coronavirus in Wuhan. MedRxiv.

[21] Diao B., Wang C., Wang R., Feng Z., Zhang J., Yang H., Tan Y., Wang H., Wang C., Liu L. (2021). Human kidney is a target for novel severe acute respiratory syndrome coronavirus 2 infection. Nat. Commun..

[22] Ye M., Wysocki J., William J., Soler M.J., Cokic I., Batlle D. (2006). Glomerular localization and expression of angiotensin-converting enzyme 2 and angiotensin-converting enzyme: Implications for Albuminuria in diabetes. J. Am. Soc. Nephrol..

[23] Moura I.B., Buckley A.M., Wilcox M.H. (2021). Can SARS-CoV-2 be transmitted via faeces?. Curr. Opin. Gastroenterol..

[24] Kim Y.I., Kim S.G., Kim S.M., Kim E.H., Park S.J., Yu K.M., Chang J.H., Kim E.J., Lee S., Casel M.A.B. (2020). Infection and rapid transmission of SARS-CoV-2 in ferrets. Cell Host Microbe.

[25] Westblade L.F., Brar G., Pinheiro L.C., Paidoussis D., Rajan M., Martin P., Goyal P., Sepulveda J.L., Zhang L., George G. (2020). SARS-CoV-2 viral load predicts mortality in patients with and without cancer who are hospitalized with COVID-19. Cancer Cell.

[26] Mezoh G., Crowther N. (2019). Deciphering endothelial dysfunction in the HIV-infected population. Adv. Exp. Med. Biol..

[27] Fan P.-C., Chang C.-H., Chen Y.-C. (2018). Biomarkers for acute cardiorenal syndrome. Nephrology.

[28] Page A.V., Liles W.C. (2013). Biomarkers of endothelial activation/dysfunction in infectious diseases. Virulence.

[29] Conway E.M. (2012). Thrombomodulin and its role in inflammation. Semin. Immunopathol..

[30] Morser J. (2012). Thrombomodulin links coagulation to inflammation and immunity. Curr. Drug Targets.

[31] Kwaifa I.K., Bahari H., Yong Y.K., Noor S.M. (2020). Endothelial dysfunction in obesity-induced inflammation: Molecular mechanisms and clinical implications. Biomolecules.

[32] Sun H.-J., Wu Z.-Y., Nie X.-W., Bian J.-S. (2020). Role of endothelial dysfunction in cardiovascular diseases: The link between inflammation and hydrogen sulfide. Front. Pharmacol..

[33] Zhou P., Yang X.-L., Wang X.-G., Hu B., Zhang L., Zhang W., Si H.-R., Zhu Y., Li B., Huang C.-L. (2020). A pneumonia outbreak associated with a new coronavirus of probable bat origin. Nature.

[34] Li W., Moore M.J., Vasilieva N., Sui J., Wong S.K., Berne M.A., Somasundaran M., Sullivan J.L., Luzuriaga K., Greenough T.C. (2003). Angiotensin-converting enzyme 2 is a functional receptor for the SARS coronavirus. Nature.

[35] Yan R., Zhang Y., Li Y., Xia L., Guo Y., Zhou Q. (2020). Structural basis for the recognition of SARS-CoV-2 by full-length human ACE2. Science.

[36] Hamming I., Timens W., Bulthuis M.L.C., Lely A.T., Navis G.V., van Goor H. (2004). Tissue distribution of ACE2 protein, the functional receptor for SARS coronavirus. A first step in under-standing SARS pathogenesis. J. Pathol..

[37] Sullivan M.K., Lees J.S., Drake T.M., Docherty A.B., Oates G., Hardwick H.E., Russell C.D., Merson L., Dunning J., Nguyen-Van-Tam J.S. (2021). Acute kidney injury in patients hospitalised with COVID-19 from the ISARIC WHO CCP-UK Study: A prospective, multicentre cohort study. Nephrol. Dial. Transplant..

[38] Marques F., Gameiro J., Oliveira J., Fonseca J.A., Duarte I., Bernardo J., Branco C., Costa C., Carreiro C., Braz S. (2021). Acute kidney disease and mortality in acute kidney injury patients with COVID-19. J. Clin. Med..

[39] Moledina D.G., Simonov M., Yamamoto Y., Alausa J., Arora T., Biswas A., Cantley L.G., Ghazi L., Greenberg J.H., Hinchcliff M. (2021). The association of COVID-19 with acute kidney injury independent of severity of illness: A multicenter cohort study. Am. J. Kidney Dis..

[40] Chan L., Chaudhary K., Saha A., Chauhan K., Vaid A., Zhao S., Paranjpe I., Somani S., Richter F., Miotto R. (2021). AKI in hospitalized patients with COVID-19. J. Am. Soc. Nephrol..

[41] Chen N., Zhou M., Dong X., Qu J., Gong F., Han Y., Qiu Y., Wang J., Liu Y., Wei Y. (2020). Epidemiological and clinical characteristics of 99 cases of 2019 novel coronavirus pneumonia in Wuhan, China: A descriptive study. Lancet.

[42] Su H., Yang M., Wan C., Yi L.-X., Tang F., Zhu H.-Y., Yi F., Yang H.-C., Fogo A.B., Nie X. (2020). Renal histopathological analysis of 26 postmortem findings of patients with COVID-19 in China. Kidney Int..

