# Supplementary material for: Urinary SARS-CoV-2 RNA Is an Indicator for the Progression and Prognosis of COVID-19
Source: Diagnostics (Basel). 2021 Nov 12;11(11):2089. doi: 10.3390/diagnostics11112089 (PMC8619898; doi:10.3390/diagnostics11112089)
Supplement: Supplementary file 1 [file diagnostics-11-02089-s001.zip › diagnostics-1412839-supplementary.pdf]

diagnostics

MDPI

Urinary SARS-CoV-2 RNA Is an Indicator for the Progression and Prognosis of COVID-19

Lu Zhang [1,†], Maoqing Tian [1,†], Yuan Song [1], Wei Liang [1], Xiaogang Li [2], Yongqing Tong [3,*], Huiming Wang [1,*]

[1] Department of Nephrology, Renmin Hospital of Wuhan University, Wuhan 430060, China; zhanglu.1@foxmail.com (L.Z.); tianmq95@163.com (M.T.); bogehers@163.com (Y.S.); Dr.liangwei@whu.edu.cn (W.L.)
[2] Division of Nephrology, Department of Medicine, Mayo Clinic, Rochester, MN 55905, USA; Li.Xiaogang@mayo.edu
[3] Department of Laboratory Science, Renmin Hospital of Wuhan University, Wuhan 430060, China
* Correspondence: tytsing@whu.edu.cn (Y.T.); rm000301@whu.edu.cn (H.W.)
† These authors contributed equally to the article and share first authorship

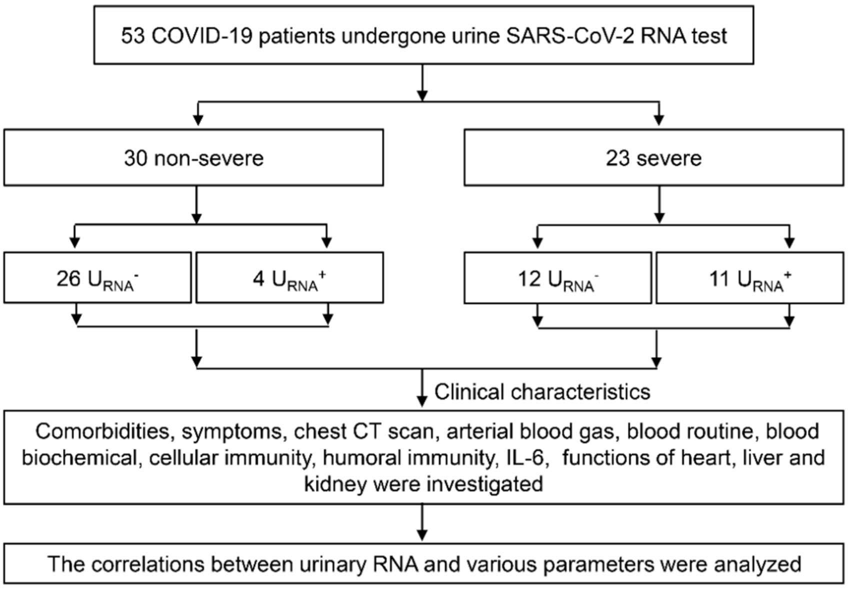

Figure S1. Study flowchart of patient's enrolling and data analysis. In this study 53 hospital COVID-19 patients who underwent a urine sediment SARS-CoV-2 RNA test at the early stage of admission were enrolled. The patients were stratified according to illness severity and urinary qRT-PCR results. Demographic and clinical features were collected and analyzed.
